# Teamwork enables high level of early mobilization in critically ill patients

**DOI:** 10.1186/s13613-016-0184-y

**Published:** 2016-08-24

**Authors:** Cheryl Elizabeth Hickmann, Diego Castanares-Zapatero, Emilie Bialais, Jonathan Dugernier, Antoine Tordeur, Lise Colmant, Xavier Wittebole, Giuseppe Tirone, Jean Roeseler, Pierre-François Laterre

**Affiliations:** Intensive Care Unit, Cliniques universitaires Saint-Luc, Université catholique de Louvain (UCL), Avenue Hippocrate 10, 1200 Brussels, Belgium

**Keywords:** Teamwork, Intensive care unit, Mechanical ventilation, Early mobilization, Physical therapy, Perception

## Abstract

**Background:**

Early mobilization in critically ill patients has been shown to prevent bed-rest-associated morbidity. Reported reasons for not mobilizing patients, thereby excluding or delaying such intervention, are diverse and comprise safety considerations for high-risk critically ill patients with multiple organ support systems. This study sought to demonstrate that early mobilization performed within the first 24 h of ICU admission proves to be feasible and well tolerated in the vast majority of critically ill patients.

**Results:**

General practice data were collected for 171 consecutive admissions to our ICU over a 2-month period according to a local, standardized, early mobilization protocol. The total period covered 731 patient-days, 22 (3 %) of which met our local exclusion criteria for mobilization. Of the remaining 709 patient-days, early mobilization was achieved on 86 % of them, bed-to-chair transfer on 74 %, and at least one physical therapy session on 59 %. Median time interval from ICU admission to the first early mobilization activity was 19 h (IQR = 15–23). In patients on mechanical ventilation (51 %), accounting for 46 % of patient-days, 35 % were administered vasopressors and 11 % continuous renal replacement therapy. Within this group, bed-to-chair transfer was achieved on 68 % of patient-days and at least one early mobilization activity on 80 %. Limiting factors to start early mobilization included restricted staffing capacities, diagnostic or surgical procedures, patients’ refusal, as well as severe hemodynamic instability. Hemodynamic parameters were rarely affected during mobilization, causing interruption in only 0.8 % of all activities, primarily due to reversible hypotension or arrhythmia. In general, all activities were well tolerated, while patients were able to self-regulate their active early mobilization. Patients’ subjective perception of physical therapy was reported to be enjoyable.

**Conclusions:**

Mobilization within the first 24 h of ICU admission is achievable in the majority of critical ill patients, in spite of mechanical ventilation, vasopressor administration, or renal replacement therapy.

**Electronic supplementary material:**

The online version of this article (doi:10.1186/s13613-016-0184-y) contains supplementary material, which is available to authorized users.

## Background

Early mobilization referring to initiating physical exercise or mobilization within the early illness phase is an increasingly common practice in intensive care units (ICU) [1]. Yet the definition of early mobilization is rather vague, as it encompasses a wide range of techniques practiced on different ICU populations [2, 3]. Nevertheless, early mobility interventions in critically ill patients prove to be feasible and safe in preventing bed-rest-associated morbidity [4–6], while improving patients’ physical function [7], psychological condition [8], and quality of life [9]. Mobilizing patients at an early time point has been associated with reduced health care costs [10], as such intervention decreases invasive mechanical ventilation (MV) duration, delirium [7, 11], and hospital length of stay [12]. Recent observations suggest that providing mobility as early as possible and extending it to weekends could further improve patient outcomes [13–15].

Reported reasons for not mobilizing patients vary widely and include mechanical ventilation [16], catecholamine infusion [17], impaired consciousness [16], poor functional status [7, 12], safety considerations [9], limited staff capacities, or lack of protocols [18–20]. Safety considerations are indeed crucial in order to prevent additional risks, yet several reported safety issues are instrumental in excluding or delaying intervention in critically ill patients on multiple support systems, whereby this group runs the greatest risk of developing neuromuscular abnormalities.

At the same time, communication [21] and muscular activity [7] remain possible by means of limiting sedation, in line with current recommendations. Nevertheless, there is a lack of data available reporting patients’ perceptions in such settings.

In our experience, early mobilization is an integral part of standard care, requiring teamwork combined with either limited sedation or none at all. The primary objective of this study was to demonstrate that early mobilization is feasible in the vast majority of critically ill patients, independently of their severity assessed by the need of MV, high FiO_2_, vasopressor doses, or renal replacement therapy (RRT). The secondary objectives included safety of early mobilization, early mobilization rate in MV according to hypoxemia severity and patients’ perception. Preliminary data were reported in an Abstract book [22].

## Methods

### Setting and patients

This was an observational study performed in a tertiary, 14-bed, mixed ICU at Saint-Luc University Hospital. Data were collected from all consecutive patients either already hospitalized in or newly admitted to our ICU between December 1, 2014, and January 31, 2015. The Ethics Committee of the Cliniques universitaires Saint-Luc, Brussels, Belgium, approved the study protocol. A waiver was obtained for written informed consent, given that the described interventions were considered to be part of standard care. Early unwanted effects of mobility, in addition to monitoring data, were anonymously recorded in accordance with Belgian and European law.

### Early mobilization and standard care

**Fig. 1:**
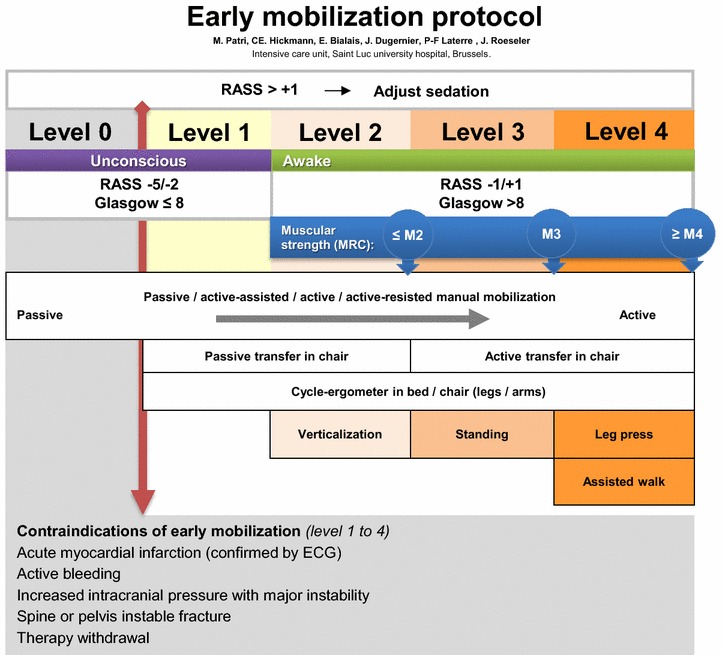
Early mobilization protocol of ICU at Saint-Luc University Hospital. Modified with authorization [24]

In accordance with the literature, we define early mobilization as a series of progressive physical activities able to induce acute physiological responses (enhancing ventilation, central and peripheral circulation, muscle metabolism, and alertness) [23] and beginning within 24 h of ICU admission. Our early mobilization protocol includes a few prior contraindications (Fig. 1) [24], such as acute myocardial infarction, active bleeding, increased intracranial pressure with major instability, unstable pelvic fractures, and therapy withdrawal. Moreover, during the morning medical rounds, a multidisciplinary team (physicians, physical therapists, and nurses) evaluates each patient in order to identify limitations to early mobilization. These include low blood pressure despite increasing dose of vasopressors, severe hypoxemia requiring a rapid increase in FiO_2_ or prone position, seizures, and patients’ refusal.

According to the routine procedure for basic treatment, ICU team first transfers patients out of their beds. The ensuing physical therapy sessions are then designed as passive, active, or manual resistance exercise; cycle ergometer or leg press training; standing; verticalization by means of a tilt table; standing and assisted walking [25]. Activities are selected depending on patients’ consciousness; hemodynamic/respiratory stability, as perceived by the team; as well as patients’ preferences and physical capabilities. The complete therapeutic regime included getting out of bed together with physical therapy sessions twice a day. The daily mobilization program is otherwise considered to be incomplete.

Physical therapists are present at the ICU from Monday to Friday (7:30 am–5:00 pm), and the senior physical therapist-to-patient ratio is 1:14. The ratio of physical therapy students to senior physical therapists is 2:1. Furthermore, one resident physical therapist is present in the hospital at all times in case of respiratory emergencies. The nurse to patient ratio is 1:1.6 from 7:30 am to 4:00 pm.

Our standard care program consists in limited sedative administration in order to keep patients dozy and calm (RASS score between −1 and +1), combined with appropriate analgesia. Our preferred mechanical ventilation mode is pressure support, irrespective of hypoxemia severity or ARDS, provided that the protective volume and pressures guidelines were adhered to [26]. Controlled ventilation modes are mainly restricted to patients undergoing prone position or very severe hypoxemia despite PEEP adjustment.

### Data collection

All medical and monitoring data were collected on a routine basis using our software of choice (*Qcare* 4.6 Build 154/2, C3 Critical Care Company NV, Sint-Martens-Latem, Belgium), with subsequent analysis performed by means of a data extraction tool. We extracted from our routine database: demographic characteristics, severity scores, monitoring data, early mobilization activities, reasons for not providing such therapy, as well as any adverse events. Predefined adverse events included death, cardiac or respiratory arrest, falls, medical device removal, and abnormal physiological responses requiring activity interruption [27].

For the first patients’ transfer to chair, the nurse monitored hemodynamic and respiratory parameters at baseline (in bed), and after 5 and 30 min, respectively. Through physical therapy sessions, hemodynamic and respiratory parameters, along with pain scores, were monitored at baseline, as well as at 0 and 15 min afterward, respectively. Pain was assessed in communicative patients on a score of 0 (no pain) to 10 (maximum pain). Patients’ perceived exertion was rated from 0 to 10 immediately following physical therapy sessions based on the Borg RPE scale [28], with a similar rating employed to measure perceived enjoyment (0 = no enjoyment, 10 = maximum enjoyment) [29, 30].

### Statistical analysis

Analyses were conducted using the software program SPSS software (IBM Corp. Released 2011. IBM SPSS Statistics for Windows, version 20.0. Armonk, NY, USA: IBM Corp). Study periods were expressed in patient-days in terms of performing early mobilization therapy or lack thereof. Descriptive statistics were conducted for demographic, clinical, and activity data and expressed as mean and standard deviation or confidence interval at 95 % (95 % CI) for normally distributed continuous variables, or as median and interquartile range (IQR) for non-normally distributed continuous variables. Categorical data were summarized using numbers or percentages. Characteristics between mobilized and non-mobilized patients were compared using unpaired Student’s *t* test or Mann–Whitney *U* test when appropriate. Categorical data were compared with Chi-squared test between groups. One-way repeated measures ANOVA was employed with time as a random factor in order to compare the effect of each activity on hemodynamic and respiratory parameters.

To clearly demonstrate the safety of early mobilization, a multivariate analysis was performed by logistic regression. Adjusted odds ratio (AOR) for 28-day, ICU, and hospital mortality was calculated as follows: Univariate logistic regression analysis was previously performed to identify every numerical instability or collinearity of different factors associated with mortalities. Validated covariates were selected to be entered into a complete multivariate logistic regression model. Variable selection was performed with a method of backward elimination, using a criterion of *p* value less than 0.20 for retention in the model. Final analysis was performed between covariates reaching a significant *p* value. Statistical tests were two-sided, and significance was set at the 0.05 probability level.

## Results

### Population description

In total, 160 consecutive patients were admitted to the ICU over a 2-month period, and 11 others were already being hospitalized at the start of the study period. The overall characteristics of the 171 included patients are presented in Table 1. The mean APACHE II score was 18 ± 7 for the entire ICU population, 20 ± 8 for mechanically ventilated patients, and 22 ± 7 for those affected by severe sepsis or septic shock. Comorbidities were present in 60 % of patients including; active cancer (32 %), end stage cirrhosis (14 %), neurologic disorders (9 %), chronic obstructive pulmonary disease (8 %), and pancreatitis (4 %). MV was provided to 51 % of patients, including 14 % with tracheostomy. Spontaneous modes, principally pressure support, were provided in 96 % of days and controlled modes in only 4 % of the mechanical ventilated population. Remaining patients had oxygenation by mask (13 %), high-flow oxygen therapy (6 %), noninvasive mechanical ventilation (1 %), or nasal cannula (21 %). The mean inspired oxygen fraction (FiO_2_) in mechanically ventilated patients was 0.46 ± 0.17. Noradrenaline was the only vasopressor administered, with a mean dose of 0.16 ± 0.23 μg kg^−1^ min^−1^. The primary sedatives employed were propofol (93 %) and clonidine (23 %). Neuromuscular blocking agents were only administered during tracheal intubation maneuvers, as necessary. Sedatives were administered to 84 % of mechanically ventilated patients. The main analgesic medications, namely opioids and paracetamol, were administrated by means of intravenous bolus, patient-controlled analgesia systems, epidural, or oral route.

**Table 1:** Descriptive patient characteristics

All admissions (*n* = 171)	Mobilized *n* = 139	Never mobilized *n* = 32	*p* value
Age^a^	59 ± 17	62 ± 17	0.36
Male^b^	80 (58 %)	18 (56 %)	0.99
SOFA score^a^	5 ± 3	8 ± 5	0.01
APACHE II score^a^	17 ± 7	22 ± 9	<0.001
Predicted mortality (APACHE II)	29 %	44 %	0.017
In-hospital mortality^b^	26 (19 %)	16 (50 %)	<0.001
In ICU mortality^b^	11 (8 %)	13 (41 %)	<0.001
28-day mortality^b^	15 (11 %)	15 (47 %)	<0.001
ICU length of stay^a^	6.4 ± 11.7	1.4 ± 2.1	0.017
Vasoactive drug use^b^	47 (34 %)	11 (34 %)	0.99
Sedative drug use^b^	68 (49 %)	13 (41 %)	0.43
Opioids use^b^	86 (62 %)	15 (47 %)	0.16
Renal replacement therapy^b^	12 (9 %)	5 (16 %)	0.32
Admission cause			
Medical^b^	74 (53 %)	15 (47 %)	0.56
Elective surgery^b^	49 (35 %)	9 (28 %)	0.54
Urgent surgery^b^	16 (12 %)	8 (25 %)	0.08

Mechanically ventilated patients (*n* = 88)	Mobilized *n* = 69	Never mobilized *n* = 19	*p* value
Age^a^	61 ± 16	66 ± 14	0.24
Male^b^	40 (58 %)	12 (63 %)	0.79
SOFA score^a^	7 ± 4	10 ± 5	0.01
APACHE II score^a^	19 ± 7	25 ± 9	0.005
Predicted mortality (APACHE II)	36 %	60 %	0.003
In-hospital mortality^b^	20 (29 %)	13 (68 %)	0.002
In ICU mortality^b^	11 (16 %)	12 (63 %)	<0.001
28-day mortality^b^	10 (14 %)	13 (68 %)	<0.001
ICU length of stay (days)^a^	10.7 ± 15.5	1.7 ± 2.6	<0.001
MV duration (days)^a^	4.9 ± 7.7	1.3 ± 1.1	0.04
Vasoactive drug use^b^	39 (57 %)	10 (53 %)	0.79
Sedative drug use^b^	58 (84 %)	13 (68 %)	0.18
Opioids use^b^	47 (68 %)	9 (47 %)	0.18
Renal replacement therapy^b^	10 (14 %)	5 (26 %)	0.30
PaO_2_/FiO_2_ ratio^b^			
>300 (*n* = 11)	10 (91 %)	1 (9 %)	0.44
201–300 (mild) (*n* = 13)	9 (69 %)	4 (31 %)	0.46
101–200 (moderate) (*n* = 42)	34 (81 %)	8 (19 %)	0.61
≤100 (severe) (*n* = 22)	16 (73 %)	6 (27 %)	0.55

Non-mechanically ventilated (*n* = 83)	Mobilized *n* = 70	Never mobilized *n* = 13	*p* value
Age^a^	56 ± 17	56 ± 20	0.96
Male^b^	40 (57 %)	6 (46 %)	0.54
SOFA score^a^	4 ± 3	5 ± 5	0.56
APACHE II score^a^	15 ± 6	16 ± 8	0.67
Predicted mortality (APACHE II)	22 %	19 %	0.69
In-hospital mortality^b^	6 (8 %)	3 (23 %)	0.14
In ICU mortality^b^	0 (0 %)	1 (8 %)	0.15
28-day mortality^b^	5 (7 %)	2 (15 %)	0.30
ICU length of stay^a^	2.2 ± 1.6	0.8 ± 0.5	<0.001
Vasoactive drug use^b^	8 (11 %)	1 (8 %)	0.99
Sedative drug use^b^	10 (14 %)	0 (0 %)	0.34
Opioids use^b^	39 (56 %)	6 (46 %)	0.55
Renal replacement therapy^b^	2 (3 %)	0 (0 %)	0.99
PaO_2_/FiO_2_ ratio^b^			
> 300 (*n* = 37)	29 (78 %)	8 (22 %)	0.22
201–300 (mild) (*n* = 22)	19 (86 %)	3 (14 %)	0.99
101–200 (moderate) (*n* = 16)	15 (94 %)	1 (6 %)	0.44
≤100 (severe) (*n* = 8)	7 (88 %)	1 (13 %)	0.99

*APACHE II* acute physiology and chronic health evaluation II score, *SOFA* sequential organ failure assessment score

^a^Values expressed as mean ± SD

^b^Values expressed as number (percentage)

### Early mobilization therapy

**Fig. 2:**
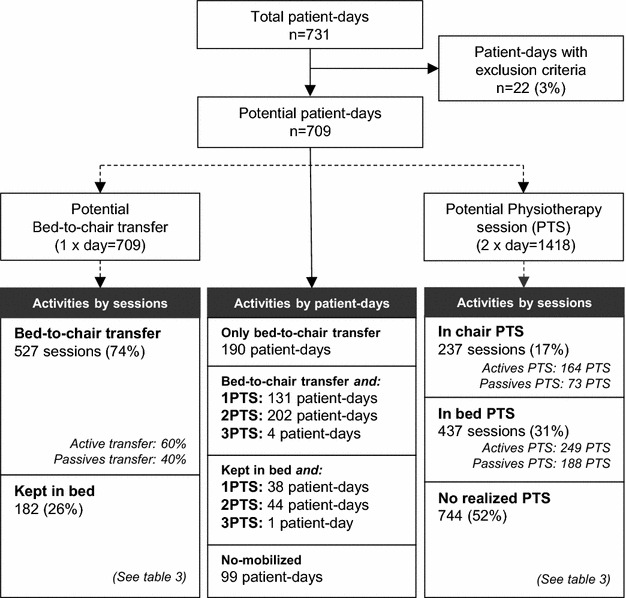
Flowchart of early mobilization activities

Overall, 139 (81 %) patients underwent early mobilization therapy. The median (IQR) delay from ICU admission to patients’ first activity was 19 h [15–23]. Seating in a chair was the first activity for 79 % of patients. In these patients, proportion of hypoxemia according to Berlin classification [31] was as follows: without (*n* = 33), mild (*n* = 19), moderate (*n* = 40), and severe (*n* = 19). The 171 ICU admissions translated to 731 patient-days. Subjects displayed protocol exclusion criteria on 3 % of patient-days. Reasons for this included active bleeding (*n* = 7), increased intracranial pressure with major instability (*n* = 3), unstable pelvic fractures (*n* = 2), and therapy withdrawal (*n* = 10). The remaining 709 were considered to be patient-days on which early mobilization was possible, thus accounting for 709 potential bed-to-chair transfers and 1418 potential physical therapy sessions (Fig. 2), according to our protocol. Based on these totals, complete and partial mobility regimes were carried out on 48 and 86 % of patient-days, respectively, and therefore incorporated into the treatment plan of 81 % of admitted patients. Subjects were transferred from their beds to chairs on 74 % of patient-days, with at least one physical therapy session provided on 59 % of patient-days.

Mobilized and non-mobilized patients’ characteristics are described in Table 2. MV, vasopressors, and RRT were provided on 46, 30, and 16 % of patient-days, respectively. Patients treated using all the aforementioned support systems were transferred out of their beds on 60 % of patient-days.

**Table 2:** Characteristics of mobilized and non-mobilized patients

	ICU patient-days	EM performed				No EM performed
		Sitting in chair			In bed PTS+	
		All sitting in chair	PTS+	PTS-		
Total	709	527	337	190	83	99
Invasive mechanical ventilation (MV)	327	223 (68 %)	142 (43 %)	81 (25 %)	40 (12 %)	64 (20 %)
Severe sepsis/sepsis shock	241	166 (69 %)	102 (42 %)	64 (27 %)	28 (12 %)	47 (20 %)
Vasoactive drugs (VAD)	211	149 (71 %)	99 (47 %)	50 (24 %)	25 (12 %)	37 (18 %)
Renal replacement therapy (RRT)	115	76 (66 %)	59 (51 %)	17 (15 %)	11 (10 %)	28 (24 %)
Sedatives (SD)	260	193 (74 %)	122 (47 %)	71 (27 %)	22 (8 %)	45 (17 %)
MV + VAD	158	104 (66 %)	72 (46 %)	32 (20 %)	21 (13 %)	33 (21 %)
MV + VAD + RRT	77	46 (60 %)	38 (49 %)	8 (10 %)	8 (10 %)	23 (30 %)
MV + without SD	122	77 (63 %)	49 (40 %)	28 (23 %)	22 (18 %)	23 (19 %)
RASS −1 to +1	576	454 (79 %)	284 (49 %)	170 (30 %)	58 (10 %)	64 (11 %)
RASS >+1	25	21 (84 %)	18 (72 %)	3 (12 %)	1 (0.4 %)	3 (12 %)
RASS <−1	108	50 (46 %)	33 (31 %)	17 (16 %)	22 (20 %)	36 (33 %)

Values expressed as number (percentage)

*MV* mechanical ventilation, *VAD* vasoactive drugs, *RRT* renal replacement therapy, *SD* sedatives drug, *RASS* Richmond agitation-sedation scale, *PTS+* physical therapy session carried out, *PTS−* no physical therapy session carried out, *EM* early mobilization

### Description of early mobilization

Patients were transferred from bed to chair with assistance in standing upright in 60 % of cases. They were manually lifted up by an ICU team in 36 % of cases, with a motorized lift employed in the remaining 4 %. Patients remained in their chairs for a median (IQR) duration of 300 (152–300) min. Hemodynamic variations during the first sitting session did not differ between patients on mechanical ventilation and those without it (Additional file 1).

Active physical therapy sessions were provided to 61 % of cases. Median (IQR) potency during active leg cycle ergometer sessions in seated and lying positions was recorded at 4 [3–5] watts and 3 [3–5] watts, respectively. Median (IQR) durations and RASS scores recorded during each activity are documented in Table 3.

**Table 3:** Early mobilization activities and patients’ perception

	Total	Duration^a^	RASS^a^	Patient perception (0–10)^b^						
				Pain				*n*	Fatigue	Enjoyment
	*n*	min	(−5 to +4)	*n*	Before	0 min	15 min		0 min	0 min
In-bed passive mobilization	151	17 [15–20]	−2 [−4 to 0]	11	4 ± 3	3 ± 3	3 ± 3	11	6 ± 3	8 ± 1
In-bed active mobilization	177	18 [15–22]	0 [0 to 0]	121	4 ± 3	4 ± 3	4 ± 3	108	6 ± 3	7 ± 3
In-bed passive cycling (legs/arms)	37	20 [15–21]	−1 [−4 to 0]	7	2 ± 3	2 ± 3	2 ± 3	7	5 ± 3	8 ± 2
In-bed active cycling (legs/arms)	69	20 [15–22]	0 [0 to 0]	64	2 ± 2	2 ± 2	3 ± 2	65	5 ± 3	9 ± 2
In-bed leg press	3	16 [10–20]	0 [0 to 0]	3	3 ± 1	3 ± 1	3 ± 1	3	5 ± 1	9 ± 1
In-chair sitting	526	300 [152–300]	0 [0 to 0]	–	–	–	–	–	–	–
In-chair passive mobilization	14	15 [12–18]	−2 [−5 to 0]	3	4 ± 4	4 ± 4	5 ± 5	1	3	5
In-chair active mobilization	41	15 [13–20]	0 [0 to 0]	22	4 ± 3	4 ± 3	4 ± 3	16	6 ± 2	6 ± 3
In-chair passive cycling (legs/arms)	59	20 [15–20]	0 [−1 to 0]	9	3 ± 3	4 ± 3	3 ± 3	4	4 ± 1	5 ± 1
In-chair active cycling (legs/arms)	93	20 [15–20]	0 [0 to 0]	74	4 ± 3	4 ± 3	3 ± 3	65	5 ± 3	7 ± 3
In-chair leg press	1	20	0	1	2	2	2	–	–	–
Standing/walking	29	28 [20–40]	0 [0 to 0]	24	2 ± 2	3 ± 3	3 ± 2	23	3 ± 2	9 ± 2

*n* Patient-days

^a^Values expressed as median [IQR]

^b^Values expressed as mean ± SD

The subjective perceptions of communicative patients were recorded on each physical therapy session (Table 3). Overall exertion ratings were moderate (5 ± 3); however, patients’ enjoyment scores following physical therapy sessions were higher, indicating pleasant perceptions of their activity (8 ± 3), with even better values observed after more demanding activities, such as walking or active cycling. It is worth noting that pain was not significantly affected by physical activity.

Hemodynamic parameters were recorded for 242 activities, 95 of which carried out by patients on MV while 147 involved no MV (Additional file 2). Heart rate, respiratory rate, or arterial pressure variations observed immediately after active exercises like walking, cycling, or manual mobilization were not clinically significant, returning to baseline values after 15 min. Hemodynamic variations on active mobilization were similar for MV and non-MV patients.

### Limiting factors for mobilization activities

Table 4 summarizes the limiting factors for early mobilization. ICU procedures (surgery, medical/nursing intervention, and imaging) were the most common reasons for patients not to perform mobilization activities, followed by physiological instability as perceived by the team, and then patients’ refusal. The failure to provide any given physical therapy session was primarily accounted for by staff limitations on weekends, and the same applies to several physical therapist consultations during the week. To a lesser extent, mobilization activities were limited due to patients’ refusal, ICU procedures, or physiological instability.

**Table 4:** Limiting factors to early mobilization

	Limiting factors to	
	Bed-to-chair transfer	Physical therapy sessions
	182 out of 709 (26 %)	744 out of 1418 (52 %)
Patient-dependent limiting factors		
Severe physiological instability	42 (23 %)	42 (6 %)
Hemodynamic instability	21	9
Respiratory instability	5	27
Neurological instability	16	6
Patient refusal	26 (14 %)	62 (8 %)
Patient-independent limiting factor		
ICU interventions	45 (25 %)	49 (7 %)
Surgery (transferred to OR)	16	16
Medical/imaging procedures	17	22
Nurse procedures	12	13
Insufficient staff (weekend)	11 (6 %)	396 (53 %)
Insufficient staff (weekdays)	0 (0 %)	16 (2 %)
No reported physical therapist consultation during week	–	177 (24 %)
Unspecified	58 (32 %)	2 (0 %)

Values expressed as number (%)

*OR* operative room

Hemodynamic instability was the most commonly reported physiological limitation to mobility, in patients receiving a mean dose of noradrenaline at 0.31 (95 % CI 0.15–0.47) μg kg^−1^ min^−1^. Noradrenaline was administered during 361 mobilization activities at a mean dose of 0.10 (95 % CI 0.09–0.11) μg kg^−1^ min^−1^. Active physical therapy was successfully performed for eight sessions, while the patients were on noradrenaline >0.2 μg kg^−1^ min^−1^ [mean dose: 0.34 (95 % CI 0.11–0.44)] and transfer from bed to chair was performed for 11 sessions in the same condition [mean dose: 0.30 (95 % CI 0.22–0.37)].

The second limiting factor was related to respiratory dysfunction on account of recent intubation/extubation (*n* = 12), prone position (*n* = 2), or occurrence of severe hypoxemia (*n* = 19). In these patients, mean FiO_2_ was 0.62 (95 % CI 0.51–0.73). Nevertheless, 78 % of MV patients were successfully mobilized with a mean FiO_2_ at 0.47 (95 %CI 0.46–0.49). We carried out 23 active and 49 passive physical therapy sessions with FiO_2_ ≥ 0.60 (mean FiO_2_ at 0.83 (95 %CI 0.77–0.88) and 0.71 (95 %CI 0.67–0.76), respectively), as well as 50 bed-to-chair transfers with mean FiO_2_ of 0.78 (95 %CI 0.74–0.82). Maximum FiO_2_ at 1.0 was observed during 18 mobility activities: nine chair sittings and nine physiotherapy activities.

### Adverse events

Activities were discontinued due to medical/nursing procedures in 11 cases and at patient request (pain, high perceived exertion, or digestive transit acceleration) in eight cases. Adverse events occurred in 10 interventions, representing 0.8 % of total mobilizations; hypotension occurred in two patients receiving low-dose vasopressors, hypertension in two, and tachycardia in three. In the sitting position, one patient experienced faintness and was subsequently diagnosed with pulmonary embolism, while another epileptic patient experienced seizures. Moreover, one patient’s operative wound exhibited slight oozing after a walking session. All events were reversible following activity interruption, displaying no impact on clinical outcome. There was no evidence of induced tissue hypoxia, as confirmed by means of steady lactate levels after mobilization available for 370 patients-days.

### Safety of early mobilization

By multivariate analyses, we were able to assess several risk factors associated with in ICU, 28-day, and in-hospital mortality (Additional file 3). Interestingly, after adjustment for severity covariates, early mobilization was not associated with increased mortality and was identified as a significant protective factor in all multivariate models (AOR (95 % CI): 0.06 (0.01–0.29), *p* = 0.001; 0.13 (0.04–0.47), *p* = 0.002 and 0.31 (0.11–0.91), *p* = 0.03 for ICU, 28-day, and in-hospital mortalities, respectively). Longer ICU length of stay, advanced age, severity of hypoxemia according to Berlin classification, and higher SOFA score were risk factors for ICU mortality. Vasoactive drug use and higher APACHE II score were risk factors for 28-day mortality. Finally, tracheostomy and higher APACHE II score were identified as risk factors for hospital mortality.

## Discussion

This observational study demonstrates the utility of teamwork in successfully carrying out early mobilization, as assessed on 171 consecutive critically ill patients. The study’s main observation is that mobility was provided at least once in 81 % of all patients within 24 h of ICU admission. Bed-to-chair transfer was achievable in the vast majority of ICU patient-days. As shown by our study data, a teamwork approach exhibited an excellent safety profile when initiated very early after ICU admission, even in patients on support by vasoactive agents, MV, or RRT. Safety of our early mobilization approach was confirmed through a multivariate analysis taking into account patients’ severity. After adjustment, early mobilization was identified not only as safe, but as a significant protective factor.

Despite the growing body of evidence confirming the feasibility, safety, and improved outcome displayed by early mobilization, it still remains a nonstandard and uncommon practice in ICUs. Moreover, initiation times vary significantly in the literature, ranging from 1.5 to 2 days [7, 32] to several days after intubation [9], or even weeks after ICU admission [33, 34]. Furthermore, several reports describe rehabilitation initiation occurring only after ICU discharge due to a lack of physical therapists or mobility teams within the ICU in question [35, 36]. In a large-scale multicenter cohort study on MV patients, mobility was achieved in only 16 % of overall sessions, reporting intubation and sedation as the primary limiting factors. In this report, authors founded a high incidence of muscular weakness and associated with higher mortality [16]. Furthermore, no clear improvement in outcome has been reported when reinforcement of physical activity was provided only after patients’ awakening [37].

Recent expert recommendations on safety criteria for early mobilization mentioned that vasopressor use [38, 39], endotracheal intubation, RRT [38], or even life support devices like ECMO [40] should not be considered as contraindications for active mobilization. Despite that, besides the study of Pohlman et al. [32] performing in-bed mobilization with maximal FiO_2_ at 1.0 and vasoactive drug, no study has explored the safety of very early mobilization in critically ill patients on multiple support systems. To date, there is no consensus regarding vasoactive doses or maximum FiO_2_, but <0.60 was considered safe for initiating active mobilization [38]. Some authors consider a maximum noradrenaline dose of 0.2 μg kg^−1^ min^−1^ and FiO_2_ < 0.55 or 0.60 to be safe [9, 38]. In the protocol at hand, we made a conscious effort to predefine a few contraindications, in order to assess each patient’s potential to undergo early activity. Our results demonstrate that mobilizing patients with higher vasopressor doses and FiO_2_ is achievable without increased risks. However, based on our data we are unable to propose theoretical limits to mobilization. Indeed, there is to our view no limiting FiO_2_ or vasopressor dose, but rather a stabilized patient’s condition with all supports.

Adverse event rates were shown to vary across studies. Pohlman et al. [32] reported the feasibility of early physical therapy and occupational therapy in 90 % of MV patients on life support devices combined with daily sedation interruption. In their study, the mean Apache II score was 20, and mobility was initiated within 1.5 days following intubation, with adverse events occurring in 16 % of overall sessions. In line with other studies, we clearly showed that most patients receiving MV and supportive therapy can be mobilized very early, within the first day of ICU admission. Furthermore, such activities were rarely interrupted due to adverse events like hypotension or arrhythmia, while requiring no additional intervention nor causing adverse outcome. We also demonstrated that mobility activities can be performed by patients following major abdominal surgery, patient that are often excluded of clinical trials.

As previously described, providing early mobilization with a high degree of supportive care requires experienced and coordinated multidisciplinary teams [41]. This is a mandatory aspect to ensure patients’ security during early mobilization implementation.

Our principal limiting factor for specific physical therapy activities stemmed from staffing capacities, resulting in 28 % of overall weekend and 12 % of weekday physical therapy activities not being performed. This likewise accounted for the low rate of walks, since emphasis was placed on less time-consuming therapies, such as ergometer cycling, in an attempt to mobilize every patient. Based on our data, we estimated the ideal ratio of senior physiotherapists to patients to be 1:7 (including on weekends) in order to achieve the optimal number of daily physical therapy activities. Furthermore, the vast majority of patients were able to be moved out of bed by the nursing team on weekends. This observation confirms that a teamwork- and protocol-driven approach is recommended in order to ensure maximum mobilization, even in the presence of a limited number of physical therapists [19]. Moreover, even if more staff is required to mobilize patients out of bed, seating patients in a chair seems to be more advantageous in the ability to achieve a greater angle of inclination and to remain in a more stable position, compared with semi-recumbent position on bed, with non-additional risks [42].

Deep sedation is usually associated with limited mobility [43]. In our study, it was therefore unsurprising to observe a lower rate of bed-to-chair transfers for patients with a RASS score <−1. Current guidelines on sedation recommend maintaining consciousness with adequate analgesia, which results in a reduction in MV duration [44], vasopressor dosage, and in-hospital mortality [45]. In line with this recommendation, RASS scores in our study primarily ranged between −1 and +1, allowing patients to communicate and self-regulate both exercise intensity and duration. In addition, patients were also allowed to refuse mobilization initiation, when expressing their inability to leave their beds or perform any physical activity. This overall approach therefore represents our optimal strategy to individually dose activity intensity and duration, coupled with vital parameter monitoring. In terms of severely ill unconscious patients, passive mobility has previously been reported to be associated with negligible variation in oxygen consumption and hemodynamic parameters [46–48].

Emerging clinical research now takes into consideration the subjective feelings of critically ill patients undergoing physical therapy in order to better dose their activities’ intensity [49]. In accordance with such methods, overall exertion values in our population were moderate, coupled with higher perceptions of enjoyment post-exercise. These observations are highly relevant for this new approach of patient-centered outcomes in critical care. Surprisingly, even during the more demanding physical activities, patients reported high enjoyment ratings.

Our study has some limitations. Firstly, this was a single-center study conducted in an ICU with a strong culture of both mobilization and minimal sedation. It may thus prove difficult to extrapolate our results to other centers. Secondly, in line with our observational study design, muscle strength or other functional outcomes were not assessed. Moreover, the protective effect of early mobilization has to be considered as an observation in our study cohort and must be confirmed by a randomized controlled trial. At last, due to the layout of the critical care units in our hospital, we did not include ischemic or heart failure patients in our study.

In conclusion, we observed that early mobilization is achievable and well tolerated in the vast majority of critically ill patients, despite commonly described contraindications such as MV, vasopressor administration, and RRT. It is of great interest to note that patients reported very positive experiences and feelings of well-being following various modalities of physical therapy sessions.

## Additional files

10.1186/s13613-016-0184-y Physiological responses during first transfer out of bed. Values expressed as mean ± standard deviation; * different from baseline, ≈ different from 5 min.
10.1186/s13613-016-0184-y Physiological responses of physiotherapy session. Values expressed as mean ± standard deviation; IB = In bed, IC = In chair, * different from baseline, ≈ different from 0 min.
10.1186/s13613-016-0184-y Multivariate analyses for risk factors associated with mortality. AOR: adjusted odd ratio; #surgery: elective or urgent surgery; * p-value <0.05.

## Abbreviations

ICU: intensive care unit
MV: mechanical ventilation
RRT: renal replacement therapy
ECMO: extracorporeal membrane oxygenation
FiO_2_: fraction of inspired oxygen
PTS: physiotherapy session
EM: early mobilization
AOR: adjusted odds ratio
