# Supplementary material for: Teamwork enables high level of early mobilization in critically ill patients
Source: Ann Intensive Care. 2016 Aug 24;6(1):80. doi: 10.1186/s13613-016-0184-y (PMC4995191; doi:10.1186/s13613-016-0184-y)
Supplement: Supplementary file 1 — 10.1186/s13613-016-0184-y Physiological responses during first transfer out of bed. Values expressed as mean ± standard deviation; * different from baseline, ≈ different from 5 min. [file 13613_2016_184_MOESM1_ESM.doc]

**Supplemental Digital Content1:** Physiological responses during first bed-to-chair transfer.

|  | **Mechanically ventilated patients (n=24)** | | | **Non-mechanically ventilated patients ( n=39 )** | | |
| --- | --- | --- | --- | --- | --- | --- |
|  | **Baseline** | **5 min** | **30 min** | **Baseline** | **5 min** | **30 min** |
| Heart Rate | 87±15 | 91±12 | 90±12 | 91±13 | 95±15* | 92±14^≈^ |
| Mean arterial pressure | 78±8 | 77±11 | 78±11 | 76±13 | 78±11 | 75±10 |
| Systolic arterial pressure | 125±17 | 121±16 | 117±18 | 116±14 | 116±19 | 112±18 |
| Diastolic arterial pressure | 63±10 | 61±12 | 65±11 | 66±12 | 66±11 | 62±10 |
| Respiratory rate | 25±6 | 27±6 | 26±6 | 25±9 | 21±4 | 22±4 |
| SaO_2_ | 95±2 | 95±2 | 96±2* | 96±3 | 95±3 | 96±3 |
| values expressed as mean ± standard deviation; * different from baseline; ≈ different from 5 min | | | | | | |
