# Supplementary material for: Teamwork enables high level of early mobilization in critically ill patients
Source: Ann Intensive Care. 2016 Aug 24;6(1):80. doi: 10.1186/s13613-016-0184-y (PMC4995191; doi:10.1186/s13613-016-0184-y)
Supplement: Supplementary file 2 — 10.1186/s13613-016-0184-y Physiological responses of physiotherapy session. Values expressed as mean ± standard deviation; IB = In bed, IC = In chair, * different from baseline, ≈ different from 0 min. [file 13613_2016_184_MOESM2_ESM.doc]

**Supplemental Digital Content2:** Physiological responses of physiotherapy session

|  | | **Heart Rate** | | | **Mean arterial pressure** | | | **Systolic arterial pressure** | | | **Diastolic arterial pressure** | | | **Respiratory rate** | | | **SaO_2_** | | |
| --- | --- | --- | --- | --- | --- | --- | --- | --- | --- | --- | --- | --- | --- | --- | --- | --- | --- | --- | --- |
| **Mechanically ventilated patients** | n | *Baseline* | *0 min* | *15 min* | *Baseline* | *0 min* | *15 min* | *Baseline* | *0 min* | *15 min* | *Baseline* | *0 min* | *15 min* | *Baseline* | *0 min* | *15 min* | *Baseline* | *0 min* | *15 min* |
| IB passive mobilizations | 19 | 98±15 | 98±15 | 99±16 | 71±11 | 75±8 | 71±10 | 114±18 | 114±17 | 112±16 | 59±9 | 64±7 | 59±9 | 26±5 | 26±6 | 25±6 | 95±3 | 96±2 | 95±3 |
| IB active mobilizations | 23 | 93±13 | 95±13 | 93±13^≈^ | 77±8 | 80±12 | 77±9 | 119±15 | 118±13 | 116±16 | 64±6 | 68±11 | 66±9 | 24±5 | 24±5 | 24±5 | 96±2 | 96±2 | 96±2 |
| IB passive cycling (legs/arms) | 8 | 100±15 | 103±15 | 100±17 | 68±7 | 70±9 | 65±9 | 104±8 | 108±11 | 102±10 | 56±6 | 59±8 | 54±8 | 27±5 | 29±4 | 28±4 | 94±3 | 93±5 | 95±2 |
| IB active cycling (legs/arms) | 5 | 87±13 | 87±9 | 82±8 | 77±5 | 81±11 | 75±9 | 128±13 | 127±19 | 119±16 | 64±5 | 68±6 | 61±10 | 21±4 | 18±4 | 15±3 | 98±1 | 97±1 | 97±2 |
| IB leg press | - |  |  |  |  |  |  |  |  |  |  |  |  |  |  |  |  |  |  |
| IC passive mobilizations | 6 | 103±18 | 99±18* | 99±18 | 70±8 | 64±6 | 68±4 | 106±8 | 97±9 | 100±7 | 60±7 | 54±6 | 59±5 | 25±5 | 27±5 | 26±3 | 95±2 | 95±2 | 95±3 |
| IC active mobilizations | 7 | 100±24 | 98±16 | 96±17 | 77±11 | 74±10 | 74±8 | 117±18 | 115±18 | 110±15 | 65±13 | 61±10 | 63±8 | 27±5 | 25±4 | 24±7 | 95±4 | 94±4 | 96±3 |
| IC passive cycling (legs/arms) | 13 | 94±18 | 94±18 | 93±17 | 72±9 | 72±7 | 74±10 | 113±12 | 111±13 | 113±15 | 61±9 | 60±6 | 63±10 | 28±7 | 27±7 | 27±6 | 92±4 | 94±4 | 93±3 |
| IC active cycling (legs/arms) | 13 | 87±13 | 89±16 | 85±16 | 73±9 | 68±13 | 73±13 | 113±15 | 108±20 | 112±19 | 62±9 | 56±13 | 62±12 | 27±5 | 27±4 | 25±4 | 93±2 | 94±4 | 95±4 |
| IC leg press | - |  |  |  |  |  |  |  |  |  |  |  |  |  |  |  |  |  |  |
| Standing /walking | 1 | 95 | 98 | 91 | 68 | 77 | 64 | 106 | 126 | 95 | 55 | 61 | 54 | 33 | 14 | 18 | 94 | 99 | 93 |
| **Non-mechanically ventilated patients** | |  | | |  | | |  | | |  | | |  | | |  | | |
| IB passive mobilizations | 9 | 99±10 | 98±9 | 99±9 | 76±12 | 67±14 | 79±7 | 115±15 | 112±8 | 115±11 | 64±14 | 65±12 | 68±10 | 20±6 | 20±6 | 19±4 | 96±3 | 96±2 | 97±2 |
| IB active mobilizations | 39 | 89±14 | 91±14* | 88±15^≈^ | 79±13 | 79±14 | 78±13 | 122±18 | 122±22 | 119±21 | 67±14 | 67±13 | 66±12 | 21±5 | 22±5 | 20±5^≈^ | 96±2 | 96±3 | 97±2 |
| IB passive cycling (legs/arms) | 8 | 86±11 | 87±9 | 85±10 | 72±8 | 75±7 | 72±6 | 115±20 | 119±20 | 116±19 | 59±6 | 63±7 | 59±5 | 19±5 | 21±3 | 20±6 | 96±3 | 96±3 | 96±3 |
| IB active cycling (legs/arms) | 23 | 92±14 | 93±14* | 91±13^≈^ | 81±11 | 81±10 | 81±11 | 124±15 | 126±15 | 123±18 | 68±11 | 67±9 | 68±12 | 22±4 | 20±3* | 20±4* | 96±2 | 96±2 | 96±2 |
| IB leg press | 2 | 110,110 | 111,118 | 109,111 | 89,90 | 96,99 | 94,99 | 128,130 | 136,142 | 132,132 | 77,99 | 83,85 | 84,89 | 28,28 | 30,26 | 26,20 | 95,97 | 94,97 | 94,97 |
| IC passive mobilizations | 2 | 97,94 | 95,94 | 94,94 | 82,97 | 95,100 | 74,96 | 105,177 | 113,176 | 104,165 | 75,73 | 82,75 | 65,71 | 26,26 | 26,24 | 27,28 | 97,96 | 97,95 | 97,95 |
| IC active mobilizations | 10 | 103±11 | 102±9 | 101±11 | 90±18 | 83±17 | 85±11 | 127±21 | 120±18 | 121±17 | 81±18 | 72±18 | 74±12 | 22±5 | 23±5 | 22±3 | 97±2 | 95±2 | 95±3 |
| IC passive cycling (legs/arms) | 9 | 96±13 | 101±18* | 98±17 | 81±13 | 80±8 | 83±13 | 115±21 | 116±16 | 119±22 | 69±13 | 69±8 | 71±11 | 19±4 | 21±5 | 18±3 | 96±3 | 95±3 | 95±3 |
| IC active cycling (legs/arms) | 38 | 94±15 | 99±16* | 94±15^≈^ | 81±11 | 84±12 | 80±14 | 119±18 | 123±19 | 119±18 | 70±10 | 72±11 | 71±13 | 21±4 | 23±6 | 20±5^≈^ | 96±2 | 96±3 | 96±3 |
| IC leg press | - |  |  |  |  |  |  |  |  |  |  |  |  |  |  |  |  |  |  |
| Standing /walking | 7 | 116±17 | 125±22* | 113±20 | 86±12 | 99±19* | 88±14^≈^ | 127±21 | 135±20 | 124±21^≈^ | 74±10 | 90±20* | 78±12 | 26±6 | 30±9 | 24±6^≈^ | 96±2 | 93±4 | 96±2 |
| values expressed as mean ± standard deviation; IB= In bed; IC= In chair; * different from baseline; ≈ different from 0 min; *n* represents patient-days | | | | | | | | | | | | | | | | | | | |
