# Supplementary material for: Teamwork enables high level of early mobilization in critically ill patients
Source: Ann Intensive Care. 2016 Aug 24;6(1):80. doi: 10.1186/s13613-016-0184-y (PMC4995191; doi:10.1186/s13613-016-0184-y)
Supplement: Supplementary file 3 — 10.1186/s13613-016-0184-y Multivariate analyses for risk factors associated with mortality. AOR: adjusted odd ratio; #surgery: elective or urgent surgery; * p-value <0.05. [file 13613_2016_184_MOESM3_ESM.docx]

ICU mortality

|  | AOR (95% CI)  *Adjusted on 15 covariables* | *p*-value | AOR (95% CI)  *Adjusted on 4 covariables* | *p*-value |
| --- | --- | --- | --- | --- |
| **Early Mobilization** | **0.06 (0.01-0.42)** | **0.004*** | **0.06 (0.01-0.29)** | **0.001*** |
| ICU length of stay | 1.10 (1.04-1.17) | 0.001* | 1.08 (1.03-1.12) | 0.001* |
| Male | 1.78 (0.27-11.63) | 0.55 |  |  |
| Age (years) | 1.09 (1.01-1.17) | 0.04* | 1.07 (1.02-1.13) | 0.01* |
| Tracheotomy | 0.67 (0.05-8.47) | 0.76 |  |  |
| Berlin classification | 1.95 (0.78-4.90) | 0.15 | 2.26 (1.01-5.03) | 0.04* |
| Surgery^#^ | 1.54 (0.50-4.69) | 0.45 |  |  |
| Cirrhosis | 2.81 (0.35-22.77) | 0.33 |  |  |
| BPCO | 0.24 (0.01-5.07) | 0.36 |  |  |
| Cancer | 1.32 (0.21-8.18) | 0.77 |  |  |
| Neurologic | 1.24 (0.06-26.49) | 0.89 |  |  |
| APACHE II score | 1.06 (0.91-1.23) | 0.49 |  |  |
| SOFA score | 1.24 (0.92-1.69) | 0.16 | 1.38 (1.14-1.67) | 0.001* |
| Sedatives drug use | 1.04 (0.07-15.33) | 0.98 |  |  |
| Vasoactive drug use | 1.99 (0.15-26.70) | 0.60 |  |  |
| Renal replacement therapy | 0.24 (0.04-1.40) | 0.11 |  |  |

AOR: Adjusted Odd-Ratio; ^#^surgery: elective or urgent surgery; *denotes p-value <0.05

28-days mortality

|  | AOR (95% CI)  *Adjusted on 16 covariables* | *p*-value | AOR (95% CI)  *Adjusted on 3 covariables* | *p*-value |
| --- | --- | --- | --- | --- |
| **Early Mobilization** | **0.11 (0.02-0.65)** | **0.01*** | **0.13 (0.04-0.47)** | **0.002*** |
| ICU length of stay | 0.99 (0.92-1.07) | 0.85 |  |  |
| Male | 2.68 (0.62-11.60) | 0.19 |  |  |
| Age (years) | 1.03 (0.98-1.09) | 0.27 |  |  |
| Tracheotomy | 0.20 (0.01-4.85) | 0.32 |  |  |
| Mechanical ventilation | 1.13 (0.14-9.37) | 0.91 |  |  |
| Berlin classification | 1.37 (0.67-2.78) | 0.39 |  |  |
| Surgery^#^ | 1.14 (0.42-3.10) | 0.80 |  |  |
| Cirrhosis | 1.65 (0.31-8.81) | 0.56 |  |  |
| BPCO | 1.10 (0.11-11.11) | 0.93 |  |  |
| Cancer | 1.16 (0.27-5.02) | 0.84 |  |  |
| Neurologic | 0.46 (0.04-5.35) | 0.53 |  |  |
| APACHE II score | 1.17 (1.03-1.34) | 0.02* | 1.21(1.10-1.32) | <0.001* |
| SOFA score | 1.14 (0.88-1.46) | 0.32 |  |  |
| Sedatives drug use | 0.27 (0.03-2.63) | 0.26 |  |  |
| Vasoactive drug use | 8.35 (1.13-61.61) | 0.04* | 6.36 (1.72-23.47) | 0.006* |
| Renal replacement therapy | 0.22 (0.05-0.93) | 0.04* | 0.02 (0.06-0.64) | <0.001* |

AOR: Adjusted Odd-Ratio; ^#^surgery: elective or urgent surgery; *denotes p-value <0.05

Hospital mortality

|  | AOR (95% CI)  *Adjusted on 16 covariables* | *p*-value | AOR (95% CI)  *Adjusted on 3 covariables* | *p*-value |
| --- | --- | --- | --- | --- |
| **Early Mobilization** | **0.35 (0.10-1.20)** | **0.10** | **0.31 (0.11-0.91)** | **0.03*** |
| ICU length of stay | 1.04 (0.99-1.08) | 0.10 | 1.03 (1.00-1.07) | 0.07 |
| Male | 2.34 (0.48-3.77) | 0.56 |  |  |
| Age (years) | 1.02 (0.99-1.06) | 0.20 |  |  |
| Tracheotomy | 5.30 (0.84-33.57) | 0.08 | 6.17 (1.20-31.62) | 0.03* |
| Mechanical ventilation | 1.09 (0.23-5.18) | 0.91 |  |  |
| Berlin classification | 1.01 (0.59-1.72) | 0.98 |  |  |
| Surgery^#^ | 1.30 (0.63-2.65) | 0.48 |  |  |
| Cirrhosis | 1.69 (0.45-6.36) | 0.44 |  |  |
| BPCO | 1.14 (0.19-6.90) | 0.89 |  |  |
| Cancer | 1.75 (0.59-5.24) | 0.31 |  |  |
| Neurologic disorders | 1.05 (0.20-5.46) | 0.95 |  |  |
| APACHE II score | 1.14 (1.02-1.26) | 0.02* | 1.21(1.13-1.31) | <0.001* |
| SOFA score | 1.13 (0.93-1.37) | 0.23 |  |  |
| Sedatives drug use | 1.16 (0.26-5.26) | 0.85 |  |  |
| Vasoactive drug use | 1.28 (0.35-4.65) | 0.71 |  |  |
| Renal replacement therapy | 0.55 (0.18-1.66) | 0.29 |  |  |

AOR: Adjusted Odd-Ratio; ^#^surgery: elective or urgent surgery; *denotes p-value <0.05
